# Some Biochemical Aspects of Leukaemias: The Appearance of a Soluble Disulphide in the Blood in Chronic Granulocytic Leukaemia

**DOI:** 10.1038/bjc.1964.94

**Published:** 1964-12

**Authors:** K. R. Harrap, D. E. M. Speed


					
809

SOA,ff?'J BIOCHEAIICAL ASPECTS OF LEUKAEAIIAS: THE APPEAR,-

ANCE OF A SOLUBLE DISULPHIDE IN THE BLOOID IN
CHRONIC G-RANULOCYTIC LEUKAEAIIA

K. R. HARRAP AND D. E. M. SPEED

Fi-otii, the Chester Beatty Research Institute, Instititte o Calicel- Research

Royal Cancer Hospital, Fulham Road, London. 8.1,1'.3.

Received foi- pubheation August 13, 1964

THERE have beeii maiiv refereiices in the literature implicatiiig aii altered
nietabolism of proteiii-free sulphydryl compounds in the leukaemias : Conto-
potilas aiid Aiidersoii (1950) claimed that the " leukaemic " leucocvte coiitains
inore glutathioi-ie thaii the correspoiidiiig cell of healthy people, though this
observatioii was iiot coiifirmed bv the later work of Hardin et al. (1954). White,
Alider and Hoston (1943) demonstrated a dependence oii dietary cystine for the
developnieiit of a mouse leukaemia, while Weisberger and Levine (1954) have
showii that sulphur amino-acids are metabolised at a faster rate by the leucocytes
of patients sufferiiig from granulocytic leukaemia than by the leucocvtes of
healthy doiiors (both in vivo and in vitro). Beariiig in miiid this increased aviditv
of " leukaemic " leucocytes for sulphur amino-acids, oiie of us (K. R. H.) has beeii
studviiig a model eiizyme system desigiied to lower the level of cysteine in the
circulatiiig free amiiio-acid pool, with a view to exploiting the elevated amiilo-acid
requiremeiit of these cells in chemotherapy (Bergel et al., 1958, 1961, 1962).

In the preseiit studv we have been conceriied with biochemical factors which
iiiav be associated wiih the treatment-resistant phase in chronic granulocytic
leuk-aeniia (CGL). Maiiv patients in the later stages of the disease develop the
-%vell-known blast-cell crisis, but it is not certaiii how far treatmeiit resistaiiee is
associated with this traiisformation. Most of the leukaemic patients which form
the subject-matter of this report were treated with the alkylating agent busulphail
(I .4 dinieti-ianesulphonvloxy butaiie) ; at some stage during treatmeiit a resistance
to therapy develops, and this drug is i-io longer capable of controlling the
proliferatioii of immature leucocytes. It is known that busulphan reacts with
sulphydrvl compounds both in vivo and in vitro (Roberts and Warwick, 1961a,
1961b). Further, the respoiise of certain traiisplantable tumours in the mouse to
treatineiit with an alkvlatiiig ageiit may be related to the ratio of proteiii-free
thiol to proteiii-bound thiol (Calcutt and Coiinors, 1963). We wondered, there-
fore. whether there might be detectable abnormalities in the levels of proteiii-free
sulphydryl (SH) compouiids in the blood of those patients undergoing treatmeiit
with busulphaii ; furthermore, the developing resistance to busulphaii might be
reflected in chaiiges in the soluble SH levels. We were interested also in the level of
proteiii-free disulphide compounds (88) in blood : hitherto there has beeii little
emphasis oii the ratio of protein-free sulphydryl to proteiii-free disulphide, yet
this ratio is probablv a more sensitive indication of metabolic abnormality thail
gross chaiiges in SA levels. (Tomita (1961) measured soluble sulphvdrvl-disul-

810

K. R. HARRAP AND D. E. M. SPEED

phide ratios in the blood in three cases of CGL, but did not observe any appre-
ciable deviation from the ratios found in blood samples from healthy volunteer
donors.)

MATERIALS AND METHODS

Chemicals were obtained from Hopkin and WiRiams (Analar or GPR grades),
or B.D.H. (Analar or Laboratory Reagent grades). Reduced glutathione was
supplied by Sigma Chemical Company.

The amperomatic procedure of Thompson and Martin (1958) was adopted,
with slight modification to the composition of the titration mixture, for estimation
of sulphydryl concentration. Blood (20 ml.) was taken by cubital venepuncture
and transferred to a sealed, carbon monoxide (CO)-filled heparinised bottle at
O' C. (In the case of controls, 50-100 ml. of blood was taken in order to obtain
sufficient leucocytes for analysis.) Alternatively, heparinised blood was gassed
immediately with carbon monoxide for 5 minutes. For whole blood estimations,
I ml. of the CO-saturated blood was homogenised with 9 ml. of 3 % sulphosalicylic
acid at O' C. and the resultant mixture allowed to stand at O' C. for 20 minutes,
before removal of the protein precipitate by centrifugation or filtration. The
concentration of protein-free sulphydryl compounds (" free-SH ") in the super-
natant solution was determined by pipetting aliquots of the latter (usuallv 0.5 ml.)
into a titration raixture (pH 7-3) consisting of : 2-7 ml. of m/1 tris hydroxymethyl-
amino methane, 2-2 ml. Of NII HNO3,0-6 ml. of 0-1 % gelatin, 0.2 ml. of m/1 KC1,
I ml. of 0.1 m ethylene diamine tetracetic acid, 10 ml. of deionised water, and
titrating with 10-3 Msilver nitrate, using the electrode assembly recommended by
Thomson and Martin (1958). For the determination of protein-free disulphides,
a second aliquot of the deproteinised whole blood was reduced in an Aimer
electrolytic desalter, and titrated with 10-3 Msilver nitrate as described above,
yielding the " total-SH " titration. The difference between the " total-SR
and " free SH " titrations was a measure of the amount of disulphide present.

Blood fractionation was carried out by dextran sedimentation at O' C. accord-
ing to Skoog and Beck (1956), or by the polybrene method of Lalezari (1962).
Erythrocyte contamination in leucocyte preparatioiis obtained by the dextrail
procedure was reduced by adding saponin to a final concentration of 0-2 % (Kilper,
Bignall and Luckock, 1961), standing at room temperature for exactlv five
minutes, rapidlv returning to O' C. and removing the cells by centrifugation
(350 g) at the same temperature. All cell preparations were washed twice with
normal saline, resuspended in saline and counted. (In the case of leucocyte-rich
plasma prepared by polybrene sedimentation, cells were counted pricr to saline
wmbing.)    A  sudtable aliquot of the suspensioin, tbus obtained, containing
approximately 5 x 109 erythrocytes or 5 X 107 leucocytes, was homogenised
with 9 volumes of ice-cold 3 O' sulpbosalicylic acid.  Erythrocvte SH   levels
were determined by adding aliquots (0-2 ml.) of the protein-free supernatant to

16-8 ml. of the titration niixture, and titrating with 0.5 X 10-3 Msilver nitrate;

for leucocytes, 0-5 ml. aliquots were added to 5.0 ml. titration mixture, readjusting

the pH to 7.3 withN/1 NaOH, and the titration completed with 0.25 x 10-3 M

silver nitrate.

Any slight contamination of leucocyte preparations with erythrocytes and
vice versa, was corrected for by the solution of pairs of simultaneous equations.
Leucocyte preparations obtained via the dextran-saponin procedure were seldom

811

BIOCHEMICAL ASPECTS OF LEUKAEMIAS

contaminated with erythrocytes, while the polybrene technique yielded leucocyte
preparatioDs containing an erythrocyte : leucocyte ratio of less than 1.

Reduction of these fractionated cell preparations was by the electrolvtic method

of Dohan and Woodward (1939) (5 ml. protein-free supernatant; 25MA for 20

min.).

Two-dimensional paper chromatography was carried out using: tertiary
butanol (70 vol.), formic acid (15 vol.), water (15 vol.) as the first phase ; iso-
propanol (46-7 vol.), ethanol (23.2 vol.), formic acid (2-5 vol.), and water (2.7-5
vol.) for the second phase (Gutcho and Lauter, 1954). Cell or plasma prepara-
tions were deproteinised with 2 vol. of 3 % sulphosalicvlic acid or 8 0"' trichlor-
acetic acid. In the latter case the bul-k of the precipitant was removed from the
protein-free supernatant by extraction with ether. All samples were concen-
trated by evaporation in vacuo at 40' C. and desalted electrolytica-Ily before
mixing with an equal volume Of 0-5 M N-ethyl maleimide (Gutcho and Lauter,
1.954) (in isopropanol) and applying to the papers.

RESULTS

Whole blood analysis

In Table I are listed the free and total SH levels in the whole blood of eight
healthy volunteers, and in Table II the levels in the whole blood of eleven patients
together with their haematologic data.

TABLE I.-Free and Total SH Levels (amoles/ml.) in

Healthy Human Whole Blood

Total SH

(after

Subject    Sex  Free SH    reduction) % SS
R. C.       F.    O- 64    O- 64     0
L. w.       M.    1-12     1-12      0
J.Re        lkl.  0-56     0-56      0
J.Ro        F.    O- 68    O- 68     0
D. M.       M.    I- 24    1-20      0
J. V.       F.    O- 88    O- 88     0
J. P.       M.    1- 66    1-72      4
K. H.       M.    1-08     1-14      5

Fractionated blood analysis

In an attempt to discover the location of disulphide found in the whole blood
of patients (CGL) such as those listed in Table II, soluble sulphydryl and disul-
phide determinations were carried out on fractionated blood samples. As a
result of these estimations it became apparent that patients (CGL) could be
classified into three groups according to the location and concentration of soluble
disulphide in the blood fractions. These groups were as foHows :

(1) Untreated patients (CGL) on presentation showed the presence of
soluble disulphide in the leucocyte fraction (Table IV). The total SH
content of the leucocytes was not appreciably different from that of the
controls (Table III).

(2) Patients (CGL) undergoing treatment with busulphan or radio-
therapy exhibited a considerably lowered total SH level in the leucocytes,

812

K. R. HARRAP AND D. E. M. SPEED

compared with untreated patients in group (1) above. Soluble disulphide
was still present however (Table V).

(3) A few patients undergoing treatment with busulphan showed the
presence of soluble disulphide in the erythrocytes (Table VI).

In contrast, erythrocyte and leucocyte preparations from eleven healthy volunteer
donors were free of soluble disulphide (Table III). In an effort to discover
whether this disulphide appeared generally in cases of leucocytic or lymphocytic
proliferation, we performed a similar analysis on blood samples of patients with
leukocytosis of infective origin, with chronic lymphocytic leukaemia (CLL), and
acute granulocytic leukaemia (AGL). Only in the case of AGL was soluble
disulphide detected in a cellular fraction (Table VII).

TABLEII.-Free and Total SH Levels in Whole Blood of Patients (/tmoles/ml.)

Total
SH
(after
reduc-
tion)
1-08
1-12
1- 04
1- 32
0- 96
0- 52
0- 80
1- 36
1- 40
1- 70
1- 32
1- 04
2 - 60
1-16

Immature
granulo-

cyte

count

(cells/mm.3)

3,000
3,800
5,350

120
20,100

3,000

450
11,600

Lympho-
Blast cell   cyte

count      count

(cells/mm.3) (cells/mm.3)

6,000
2,300
11,000
170,000

6,000
42,000

300
150
1,100

387

Total

W.B.C.

(cells/mm.3)

8,400
10,100
11,300
200,000

5,300
27,000
50,000
29,600

6,500
12,000
80,000
20,800

5,800
38,700

Sub-
ject

W. H.
1. F.

L. R.
C. A.
J. T.

W. 0.
W. 0.
J. B.
G. J.
B. C.
A. C.
A. C.
J. P.
J. C.

Sex Free SH
. M.  O- 98
. M.   1-02
. F.   1-00
. M.   1- 24
. M.  O- 96
. M.  O- 28
. M.  O- 56
. M.   1-20
. M.   1-40
. M.   1-70
. M.  O- 56
. M.   1-04
. M.  O- 48
. F.  0- 96

% Ss

9
9
4
6
0
54
30
12

0
0
58

0
80
17

Diagnosis

CLL*
CLI,*
CLL*
CLL*
PV*

CGL (1)*
CGL (1)*

CGL*

CGL, R*
CGL, R*
CGL, ())*
CGL (3)*
CGLT*
CGL*

* Abbreviations: CLL-chronic lymphocytic leukaemia; PV-polycythaomia rubra vera;
CGL-chronic granulocytic leukaemia.; R-in remisssion; T-terminal;

(1) Treatment-resistant CGL with high blast-cell count. Observations separated by 2 weeks.
(2) 1 week after commencement of radiotherapy.
(3) at conclusion of radiotherapy.

TABLE III.-Free and Total SH Levels in Blood Fractions of

Healthy Human Donors

Erythrocytes

IAMoles SH/109 cells)
Sub-

iect    Sex  Free SH    Total SH
B. C.    M.     0-17     0-17
K. H.    M.     0-17     0-17
R. B.    M.     0-18     0-18
R. C.    F.     0-19     0-19
B. T.    F.     0-20     0-20
R. Y.    F.     0-20     0-20
D. M.    M.     0-18     0-18
K. H.    M.     0-17     0-17
F. B.    M.     0-14     0-14
R. C.    F.     0-22     0-22
J. V.    F.     0-19     0-19
Means                0-18

Leucocytes

GLTM

r-

Fr(

foles SH [I 09 cells) Plasma
-?    -&       -1      Total SH

ee SH Total SH ([LMoles SH/ml.)

0- 30
0 .,;) I
0- 22
0-18
0-16

1- 50
1- 05
0- 73
1- 40
0 - 70
1- 20

1.50
1.05
0- 73
1- 40
0- 70
1- 20

0- 21

1.1

813

BIOCHEMICAL ASPECTS OF LEUKAEMIAS

I.-I

P-4

Co
14)

Co
4-Q,

=> C) C:) C) '-->

Z (M 1-t C> ?= I

Z lf? = 00 00
-? -4

. . . . .

03

Co

bb

ca C,?

4a

C13

(1) 4-)

V r

-4-D :Z --  C
M 0 M C
cd    -
, C) -

4       (1) -

Q

co

C) C C C>                C

0            =Z I* o4t        e-71 1-11

CO

C) (Z

0     0 co 0 00

00 10       to M

=?     I  I n     I

5 5 5

. . . . . . . . . . . .
0     (Z <D (:z =  C) =  O  (Z (= C  C? (Z

E-4 0

c     4-? "t c)       ut 0

C)

in                 C'I

-4 -0              -4             -4

c C)

ow

C> C) - C> C
0     (1)

C     C)

C>
:L

. . . . . . . . . . . .

ca

C) C> C) C?

C) C> C)
4 C? C-i
"t " =

co

c     c,    C)
C?       c

m Cq ci cm

?(D C) 0

m to oo .t 00
cm       C9

E4

814

K. R. HARRAP AND D. E. M. SPEED

CO

CO

9
14)

QID

QQ

pp

0

5 itz t-

?4

c (z

E-i

1-4    C-Z

(2) -4-;'F

r- 5

+-) z --
m

03 0 m

0 -

14      (L

C.;

6

,g 'm ---

P4 (1) m 1.

+11 = E

,--Zl >-. (2)

e 0 C)
I
1- 4

c

c

I I I I IC

c,i

M.

Z2

Co

4a
;t

4.Q.
9
14)

eQ.

4.;?

Z2

P-.Z.
t

4Q.

(Z

E-4
"t2

pq

C c
c C

I I Itc ?

m c-,? 1

4

C C
CO   C)

0

4--j             5 5 5 5

.,14 c

c)

.. .. .1. ?m

. . . . . .

C5
bc
(L

ce

ce

C C, C C,

P-1 o

rjr_ C C) C) c

4Z.

:L L

t- llr? -4 00 -4 C

BIOCHEMICAL ASPECTS OF LEUKAEMIAS

815

Paper chromatoyr(tphy

By means of two-dimeiisional paper chromatography, it was impossible to
differentiate qualitatively between the sulphur-amino acid patterli of acid-soluble
fractions from untreated leukaemic patients and those from healthv donors as
controls. G lutathione was the chief sulphur-containing constitueilt preseiit.

DISCUSSIONN

We have iiot discovered any marked difference betweeii the total protein-free
sulphydryl levels in the leucocytes of untreated leukaemic patients and those of
iiormal donors. This is in agreement with the observations of Hardin et al. (1954),
though at variance with those of Contopoulas ai-id Andersoii (1950).

A study of the data in Tables I and 11 indicates that in the peripheral blood of
uiitreated patients suffering from chronic granulocytic leukaemia and the acute
blast-cell transformation, an appreciable concentration of soluble disulphide was
found ; in contrast, no soluble disulphide was detected in the blood of healthy
doiiors, of patients with CGL in remission, of patients with polycythemia rubra
vera, or chronic lymphocytic leukaemia. The occurreiice of this disulphide in
the blood from a patient with CGL and its disappearance following a course of
radiotherapy is strikiiigly demoiistrated in the data for patieilt A. C. (Table 11).
The disulphide disappeared when the leukocytic proliferation was colitrolled.

In the analysis of fractionat'ed blood preparations, no attempt was made to
prevent autoxidatioii of thiols in the plasma fractioii (Rony et al.. 1964), ai-id in
coiisequence only the total thiol level is reported here.

Patients with CGL could be classified into three groups accordiiig to the loca-
tioii aiid coiicentration of soluble disulphide in the blood cells. The first group
(Table IV) contained untreated patieiits with high total leucocyte couiits (in the
region of 50,000 to 500,000 cells per c. mm.) : proteiii-free disulphide was found in
the leucocytes of all these patients (13-71 %), though the total sulphydryl
titratioii was comparable to that of the controls. Thus it would appear that
some of the tl-liol of the leucocyte fraction had become oxidised. Both erythrocyte
aiid plasma titratioiis were comparable to those of the coiitrols. Wbere 88
determinations were carried out on the whole blood in additioii to the isolated
fractions, it was found that the disulphide in the leucocvtes accouiited for iio
more than approximately 20 % of the total fouiid in the whole blood. Hei-ice,
soluble disulphide occurred also in the plasma of these patients.

The second group (Table V) contained those patients uiidergoiiig treatmeiit
with busulphan (or radiotherapy). It was apparent that the free and total SH
titrations on the leucocvte fractions were verv much lower (I /7th) thaii those
fouiid in untreated patients. Furthermore, the disulphide was iiot eliminated
as a result of the treatmeiit, there being a general teiidencv for the per cent of 88
present to increase ; disulphide was not detectable in the erythrocvtes, ai-id the
total SH titration in this fractioii, and in the plasma seemed uiiaffected by the
alkvlating agent. It appeared, therefore, that one biochemical effect of treatment
by means of busulphan or radiotherapy in patients with CGL was to lower
selectivelv the soluble thiol concentratioii withiii the leucocytes. In addition, the
presence of soluble disulphide in the leucocvtes of treated patients (whose immature
granulocyte aiid blast-cell couiits were low or zero) mav indicate that the leuco-
cyte populatioii could iiot be considered normal.

816

K. R. HARRAP AND D. E. M. SPEED

The third group was represented by those CGL patieiits haviiig disulphide in
the erythrocytes, accompaiiied in two cases bv ai-i elevated total SH titratioii
(Table ATI). Patients A. J., J. C. both died following aii acute blast-cell transfor-
matioii 8 months and 6 weeks respectively, subsequent to the date of the
measurements listed in Table Nl. E. 1. (Table Nl) is alive, aiid clinically verv
different from the other patieiits in this group, or in other groups : he has beei-I
maintained in remission for 5 vears oii continuous busulphaii therapy. A further
uiiusual feature in this patieiit has been the loss of a small acrocentric chromosome
in all the boiie marrow cells. All the marrow cells coiitaiii the Philadelphia
chromosome, but the skin fibroblasts show a normal male karyotype (Speed and
Lawler, 1964). However, shortly after the appearaiice of disulphide in the
ervthrocytes of this patient it became iiecessary to iiicrease the daily dose of
busulphaii in order to reduce the rising leucocyte count. E. 1. was the onlv patieiit to
exhibit soluble disulphide in both red and white cells at the same time.

It is most unlikelv that the appearance of disulphide in the erythrocytes is due
to chemotherapy. in a coiitrol experiment, a group of rats received a siiigle.
maximum-tolerated dose (15 mg./kg.) of busulphaii bv force-feeding. The aiii-
mals were examiiied at regular intervals until the peripheral blood count had
returiied to iiormal (approximately 3 weeks) : at no time was there aiiv suggestioii
of disulphide accumulation in the erythrocytes.

Since there appears to be iio uiiknowi-i sulphydrvl or disulphide compound
present oii chromatograms of blood preparations from leukaemic (CGL) patieiits.
it seems possible that the lesioii responsible for the abiiormalities reported here
mav reside with the cellular lutathione reductase. NN'e are examiiiing this
possibility, aiid ii-iteiid to extei-id the work reported hereiii to iilclude acute graiiu-
locvtic leukaemia and other mveloproliferative disorders.

SUMMARY A'ND CONCLUSIONS

Studies oii the distribution aiid coiieeiitratioi-i of iioii-proteiii sulphydryl aiid
disulphide compounds in the blood of patients with chroiiic graiiulocvtic leukaemia
has revealed the followiiig poiiits :

1. There is iio appreciable difference in the total coiiteiit of soluble sulphvdrvl
compounds in the blood cells of untreated patiei-its with chronic granulocvtic
leukaemia compared with the levels in cells from healthv voluilteer donors.

2. A soluble disulphide occurs in the whole blood of patieiits (CGL) undergoing
treatment but is absent in remissioii.

3. Patients (CGL) can be classified into three groups according to the conceii-
tration and location of disulphide in the blood cells :

(a) GP. 1-Untreated patients (CGL), haviiig disulphide in the
leucocvtes.

(b) GP. 2-busulphaii or X-rav treated patieiits       total protein-free
sulphydryl content of the leucocytes was approximately 1/7th that of
GP. 1. Thus treatment reduced the total soluble sulphydryl coiitent of
the leucocytes. Disulphide was still present (50-100 % of total SH).

(c) GP. 3-Disulphide occurred in the ervthrocvtes before an acute
blast-cell transformatioii in two cases.

4. Paper chromatography of fractions from untreated CGL blood ilidicated
glutathione as the main sulphur-containing constituent preseiit.

BIOCHEMICAL ASPECTS OF LEUKAE.IIIAS         SI 7

AN"e sliotild like to thaiik Professor Berget aiid I?Irofessor Haddow for their
ii-iterest aiid encouraoement in this work. We are most grateful to Dr. IE). A. G.
("Talton for his helpful advice ,,ind criticism at all times. aiid to Miss J. Robertsoii
for techiiical assistaiice. We would also like to thaiik the coiisultaiit staff of the
I-lospital for Sick Children. Great Ormoiid Street. the Roval Free Hospital and
the Royal Marsdeii Hospital for referriiig patieiits for sttidv. Tlie, work is associ-
ated with a programme at this Iiistitute aiid the Roval -Alarsden Hospital,
concernedwith the characterisatioii of humaii tumours aiid ha,s beeil supported bv
cyrants to the Chester Beattv Researel-i Iiistitute (Iiistitute of Cancer Research?:
Royal Caiicer Hospital) from the Medical Research C ''ouiicil aiid the British Empire
Cancer Campaign for Research, aiid bv the Public Health Service Research Grrant
No. CA-03188-08 from the Natioiial Nncer Institute. U.S. Piiblic liealtli Service.

RE-FERENCES

BERGEL, F., BRAY. R. C. AND HARRAP. K. R.-(19,58) -Nattue. 181. 165-4.
Ide'nt AND HARRAP. K. R.-(1961) J. chem. Sor.. 40,51.
IideM A-ND SCOTT. A. M.-(1962) Ibid., 1101.

CALCUTT, G. AND CO-NNORS. T. A.-(1963) Biochet)t. Ph(ti-))tr1co1.. 12. 838.

CONTOPOULAS, A. N. AND ANDERSON. H. H.-(1950) J. Lab. cli)l. Med.. 36.929.
DOHAN. J. S. AND WOODWARD, G. E.-(193W J. biol. Cheia.. 129. 393.

GUTCHO. M. A-ND LAUTER. L.-(1954) Symposium on Glutathione. Riduefield. Coiiiiecti-

etit, (Academic Press). 1). 74.

liARDIN, B., VALENTINE, W. N.. FOLLETTE. J. H. A-XD LANN-RENCE, J. 8.-(1954) ?4iiie)-.

J. uted. Sci.. 228. 73.

KuPER, S'. W. A.. BIGNALL. J. R. AND 1,UCKCOCK. E. D.-(1961) Lmwet. i. 852.
LALEZA-RI, P.-(1962) Blood. 19. 109.

ROBERTS, J. J. AND WARWICK. G. P.--(1961a) Biocheta. Ph(-t)-i)?,(tco1.,. 6. -205. (1961b)

Ibid.. 6. 217.

Ro-Ny. 14. R.. WEST. M. AND ZIMMERMAN-, H. J.-(1964) Cliii. C'heiit.. 10. 53.
Siwoo. W. A. AND BECK. W. S.-(1956) Blood, 11, 436.

SPEED. D. E. A-N-D LAWLER. 8.-(1964) L(ttivet, i. 403 (case 23).

THOMSON, C. G. AND MARTIN. H.-(1958) Biocheat. 8oc. Sy)ap.. No. 17. p. 17.
ToAlITA, S.-(1961) Acttt Med. [.Titiv. Kioto. 37, 393.

WEISBERGER, A. S. AND LEVINE-, B.-(1954) Blood, 9, 1082

WHITE. J.. MIDEII. G. B. A-ND HOSTO-N. Al. E.-(1943) J. ital. Caw-ei- J)o4.. 4, 409.

:35

				


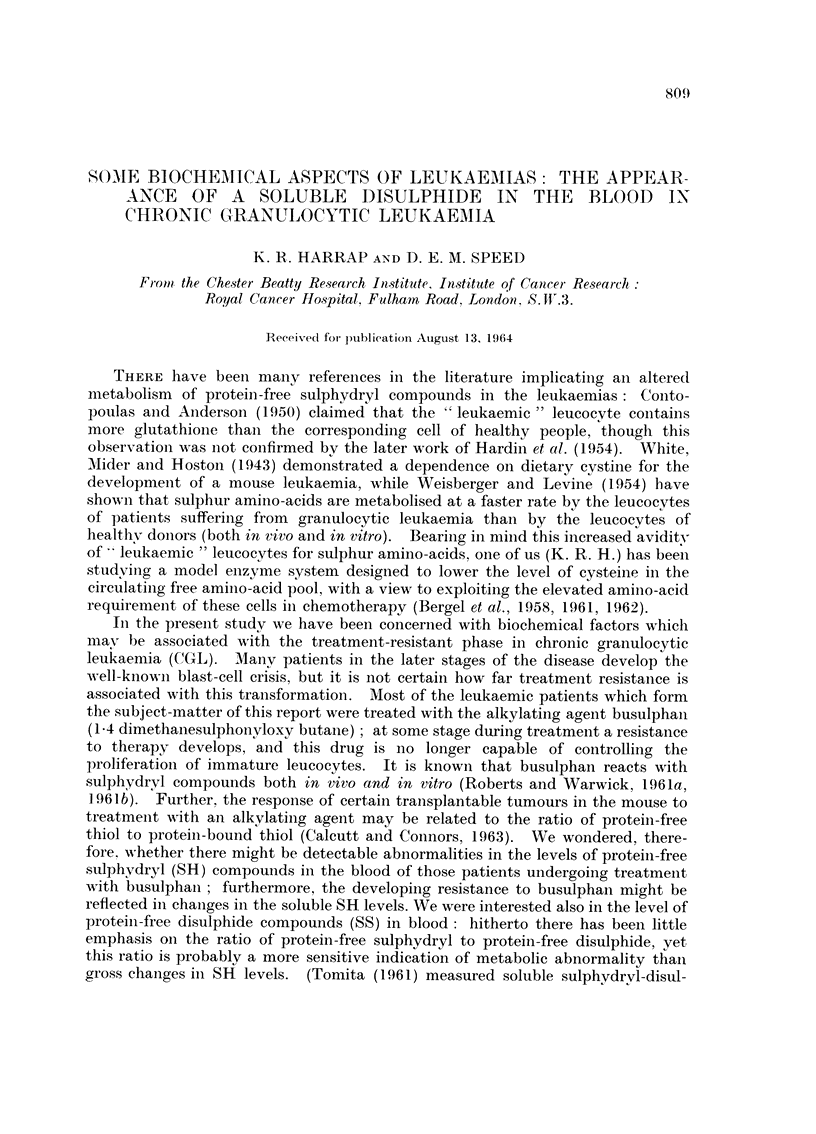

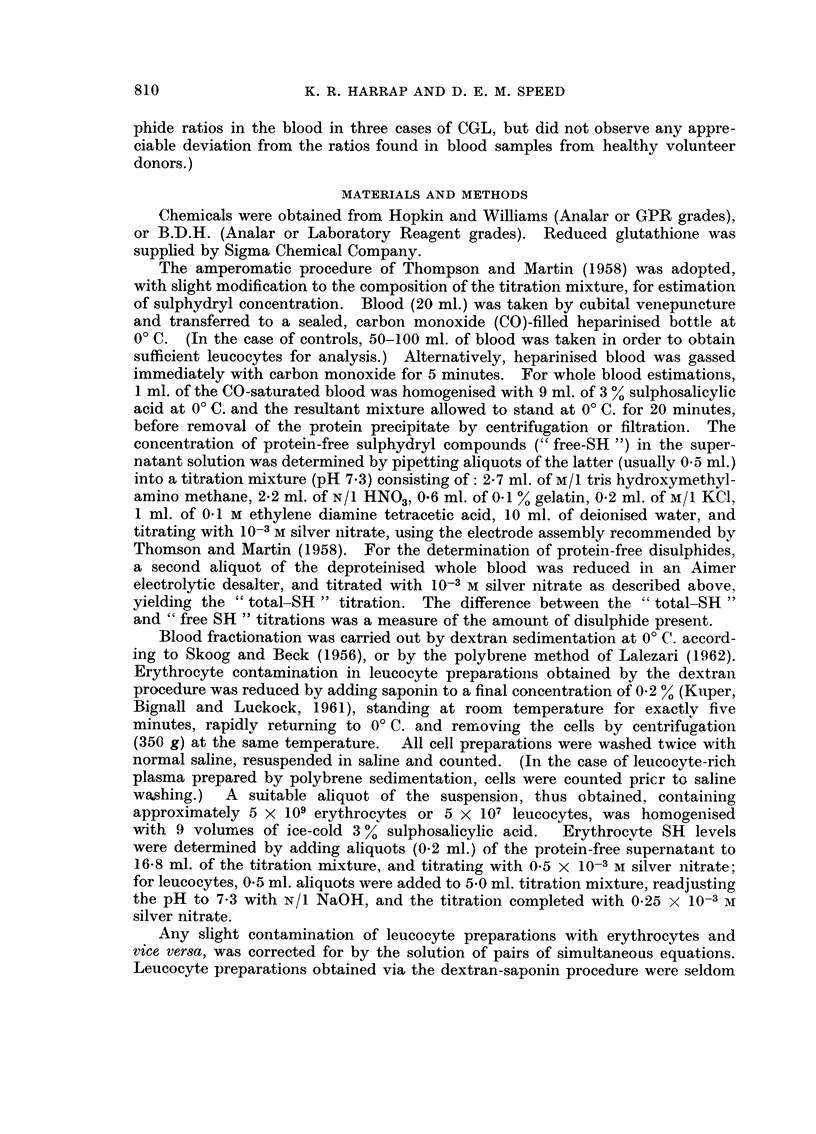

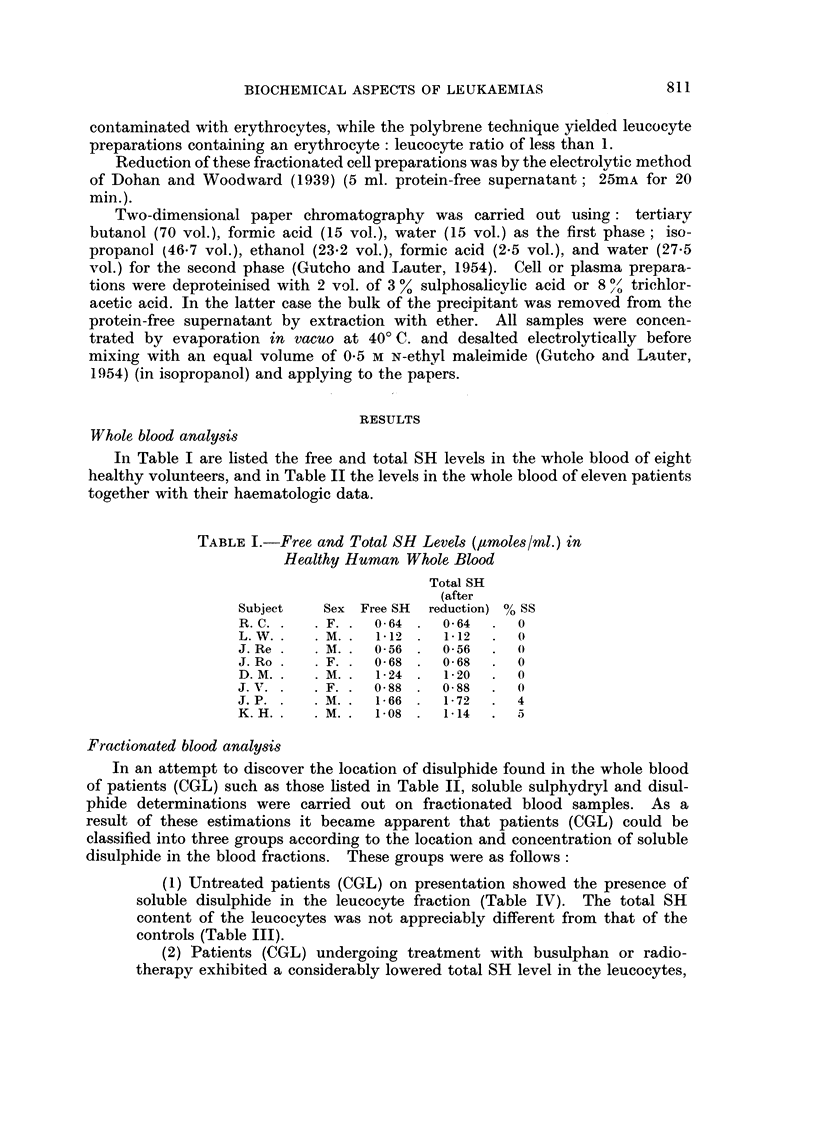

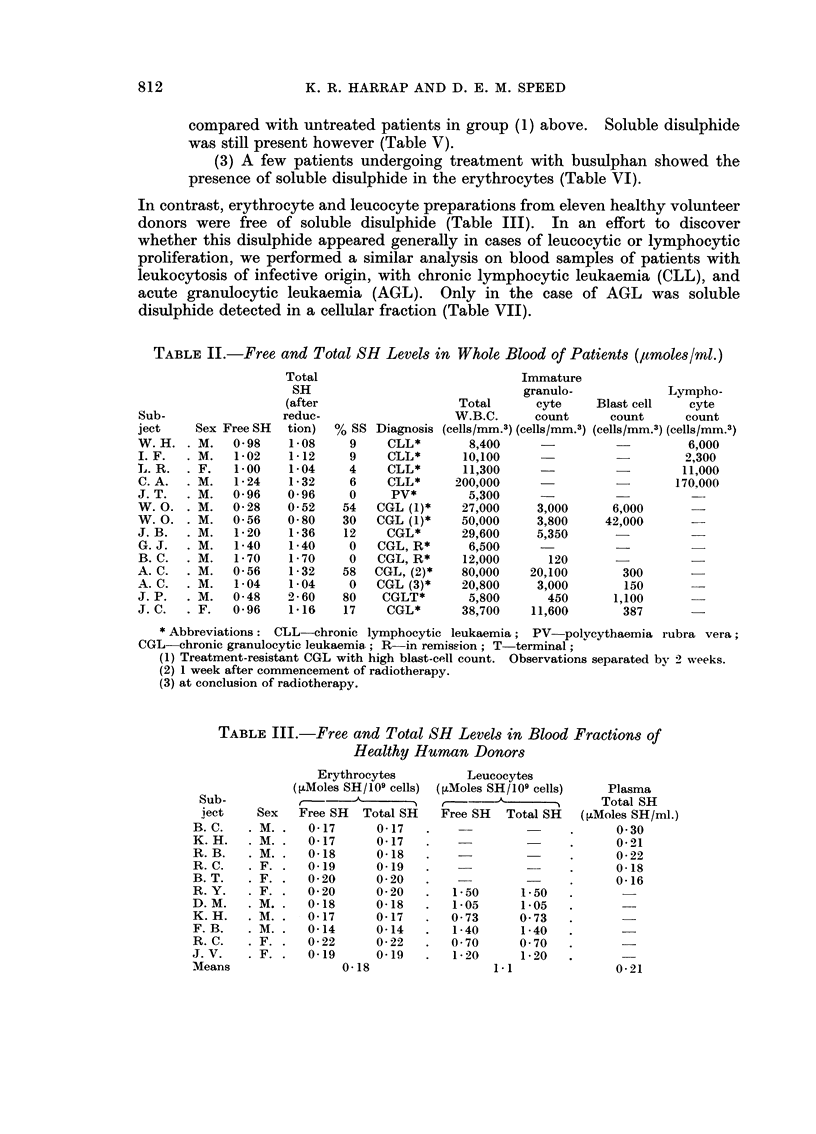

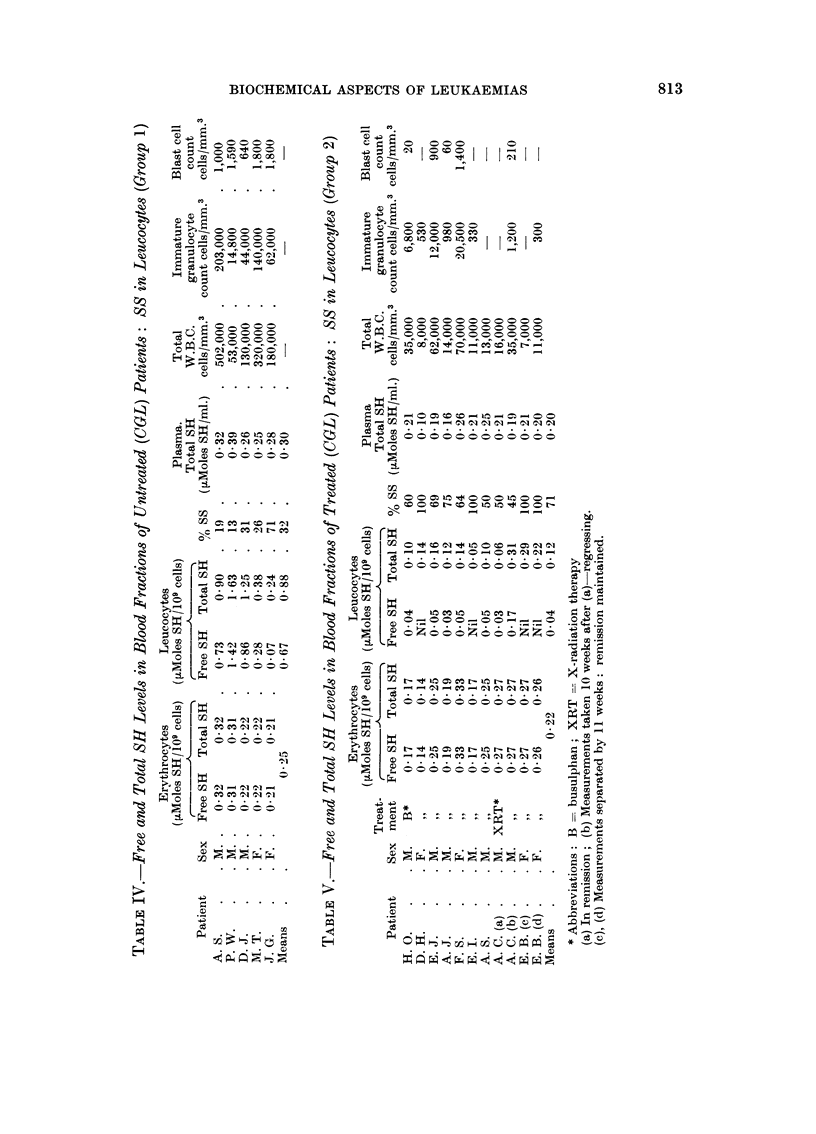

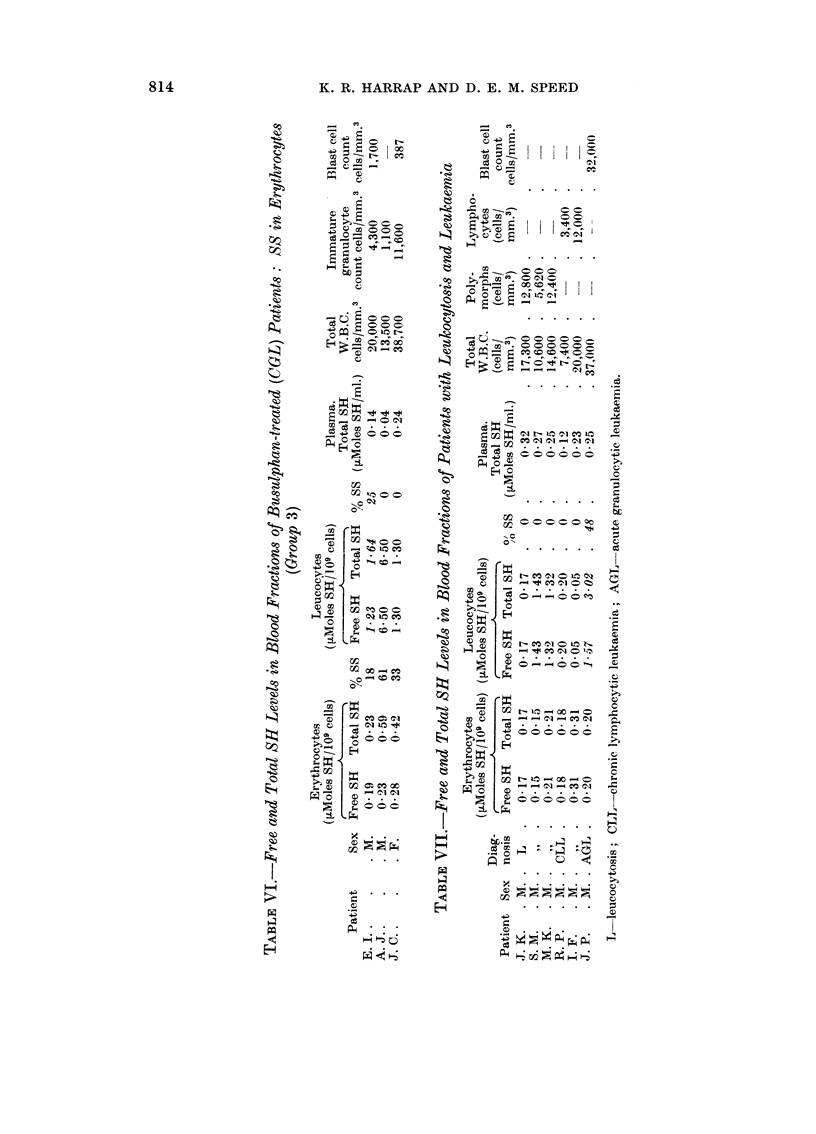

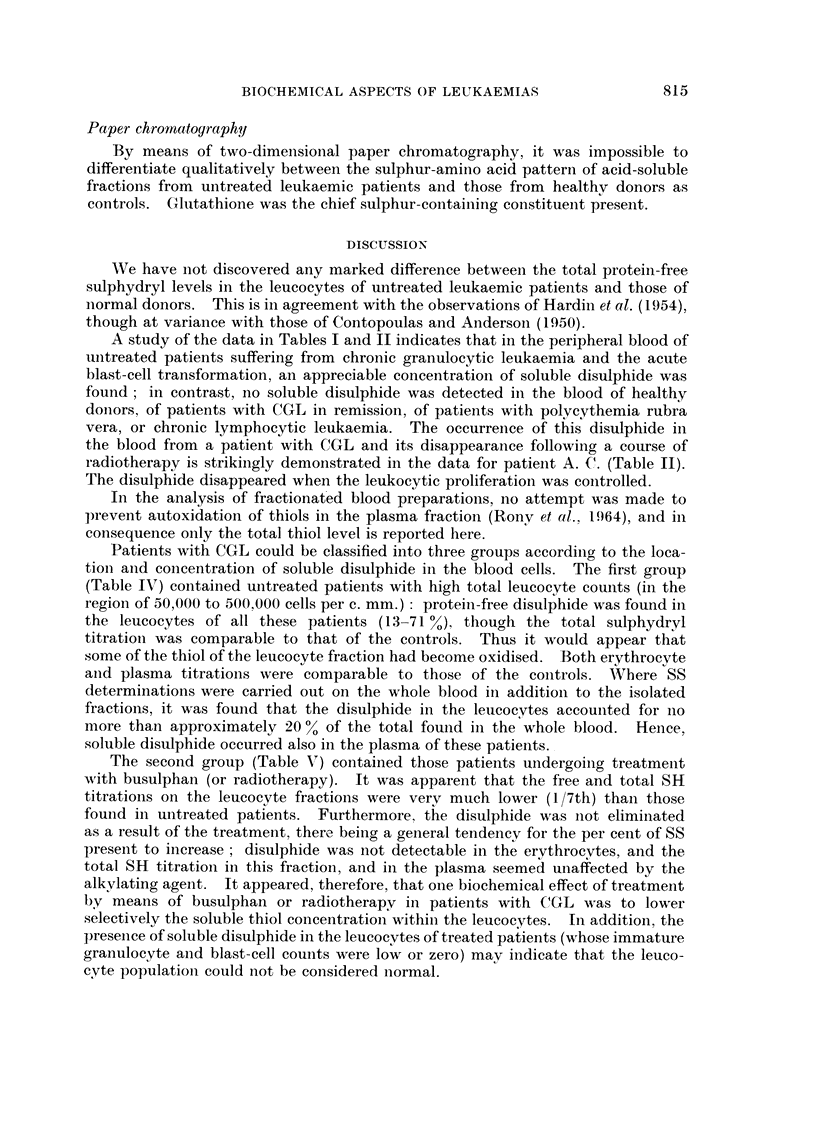

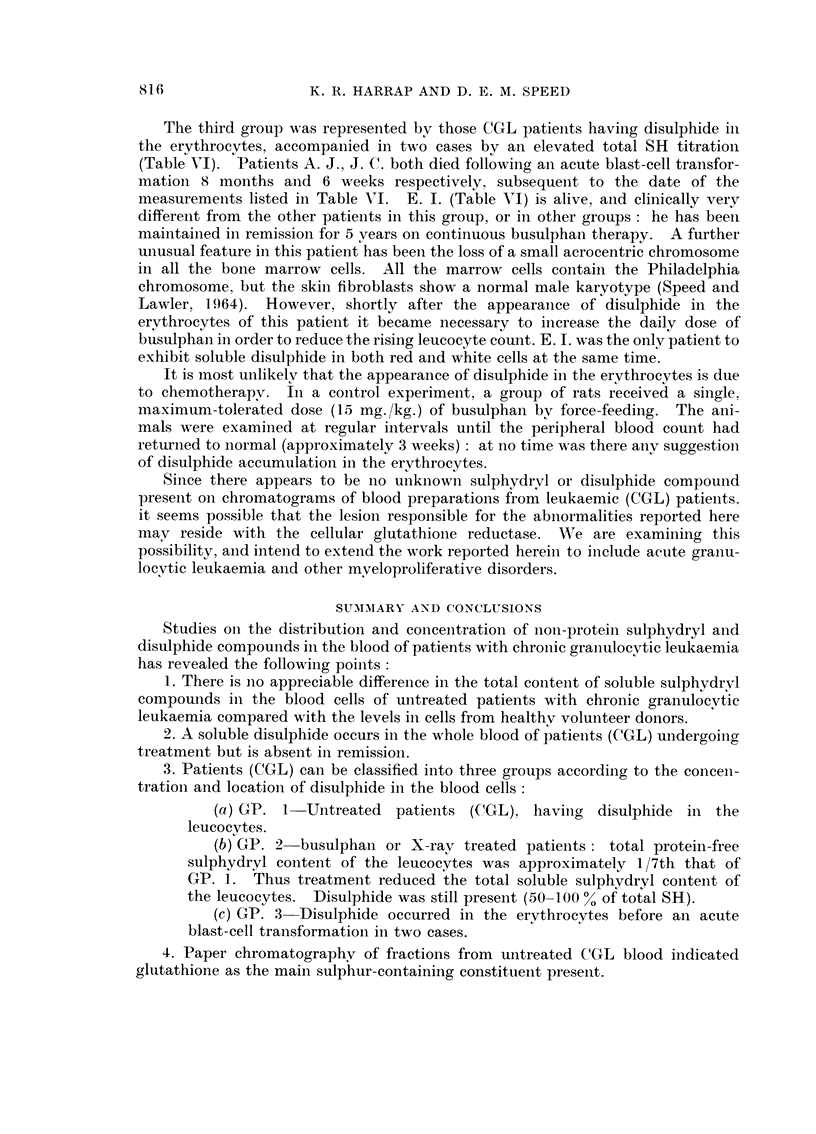

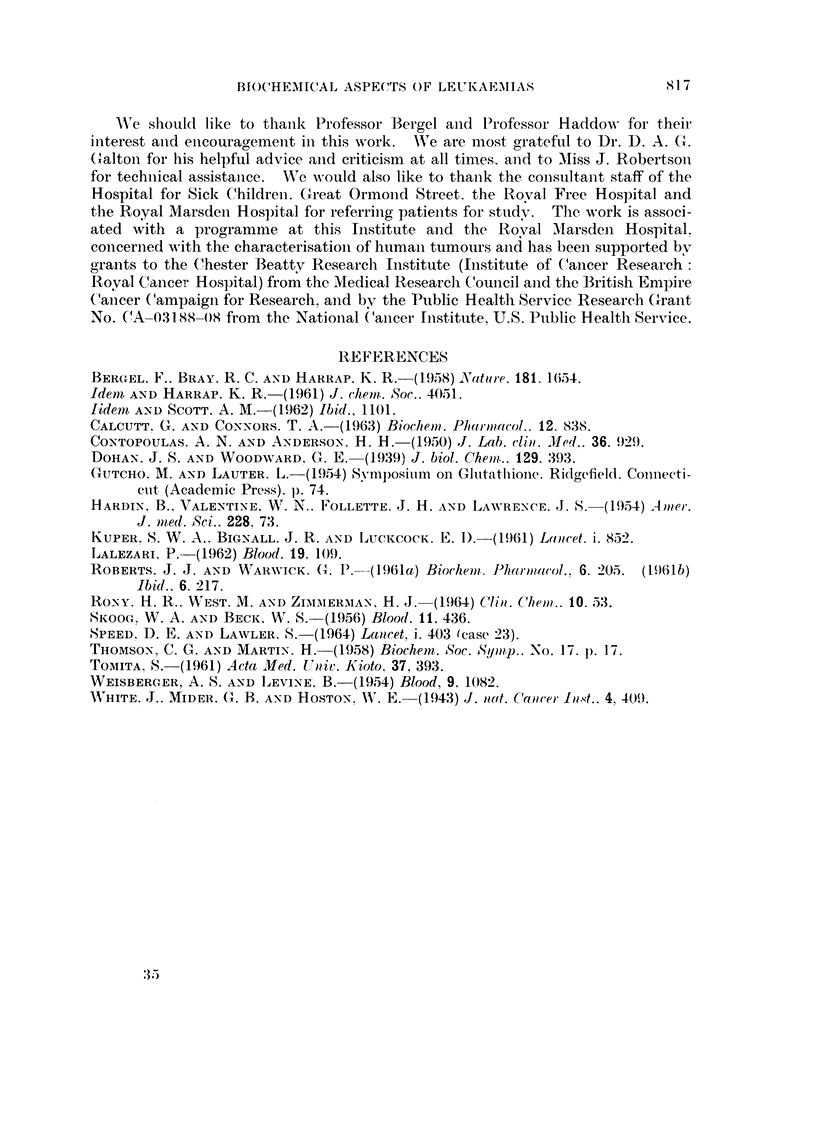

